# Efficacy of CDK4 inhibition against sarcomas depends on their levels of CDK4 and p16ink4 mRNA

**DOI:** 10.18632/oncotarget.5829

**Published:** 2015-10-20

**Authors:** Marco Perez, Sandra Muñoz-Galván, Manuel P. Jiménez-García, Juan J. Marín, Amancio Carnero

**Affiliations:** ^1^ Instituto de Biomedicina de Sevilla, IBIS/Hospital Universitario Virgen del Rocio/Universidad de Sevilla/Consejo Superior de Investigaciones Cientificas, Seville, Spain; ^2^ Department of Public Health and Preventive Medicine, University of Seville, Seville, Spain

**Keywords:** cell cycle, CDKs, cyclin dependent kinase inhibitor, sarcomas, cellular senescence

## Abstract

Sarcomas are malignant tumors accounting for a high percentage of cancer morbidity and mortality in children and young adults. Surgery and radiation therapy are the accepted treatments for most sarcomas; however, patients with metastatic disease are treated with systemic chemotherapy. Many tumors display marginal levels of chemoresponsiveness and new treatment approaches are needed. Deregulation of the G1 checkpoint is crucial for various oncogenic transformation processes, suggesting that many cancer cell types depend on CDK4/6 activity. Thus, CDK4/6 activity appears to represent a promising therapeutic target for cancer treatment. In the present work, we explore the efficacy of CDK4 inhibition using palbociclib (PD0332991), a highly selective inhibitor of CDK4/6, in a panel of sarcoma cell lines and sarcoma tumor xenografts (PDXs). Palbociclib induces senescence in these cell lines and the responsiveness of these cell lines correlated with their levels of CDK4 mRNA. Palbociclib is also active *in vivo* against sarcomas displaying high levels of CDK4 but not against sarcomas displaying low levels of CDK4 and high levels of p16^ink4a^. The analysis of tumors growing after palbociclib showed a clear decrease in the CDK4 levels, indicating that clonal selection occurred in these treated tumors. In summary, our data support the efficacy of CDK4 inhibitors against sarcomas displaying increased CDK4 levels, particularly fibrosarcomas and MPNST. Our results also suggest that high levels of p16^ink4a^ may indicate poor efficacy of CDK4 inhibitors.

## INTRODUCTION

Sarcomas are malignant tumors derived from the mesenchymal nonepithelial tissue developed from the embryonic mesoderm [1], comprising less than 10% of all cancers [2, 3] but account for a higher percentage of overall cancer morbidity and mortality in children and young adults than in adults. The overwhelming majority of sarcomas are sporadic with unknown etiology, but several well-described genetically linked cancer predisposition syndromes and well-documented types of environmental exposure have been associated with specific types of sarcoma [2, 3]. Sarcomas arise from multiple lineages and range from indolent to highly invasive and metastatic [4, 5]. Taxonomical analysis of sarcomas has identified approximately 60 subtypes of sarcoma, as well as more than 50 benign tumor subtypes [4]. Sarcomas are usually grouped in two broad categories according to molecular genetics: sarcomas harboring a diploid or nearly diploid karyotype and simple genetic driver alterations, such as Ewing's sarcoma, or sarcomas with a complex and imbalanced karyotype, such as osteosarcoma. Both subgroups include very different clinical entities and are broadly drawn, not reflecting the genetic diversity among tumors of a given type or subtype or their diverse tumor biology [4].

Surgery is the accepted treatment for most sarcomas. However, for those patients with unresectable disease or residual tumors following surgery, radiation therapy is also used. Patients with metastatic disease are treated with systemic chemotherapy, usually consisting of doxorubicin and ifosfamide [6]. This approach has been demonstrated to be effective in patients who have localized tumors, and the long-term survival rates of such patients are increasing [6]. However, for many tumors that exhibit marginal levels of chemoresponsiveness and for metastatic disease, new treatment approaches are needed. Treatments for sarcomas include doxorubicin, gemcitabine, ifosfamide and the recently accepted drug trabectedin [7–10]. Currently, few specific genetic alterations in sarcomas are direct targets for therapy, in contrast to the circumstance for epithelial cancers. The exceptions are GISTs, in which the Kit kinase inhibitor imatinib induces a partial response or stable disease in approximately 80% of sarcoma patients [4]. Those findings support the hypothesis that widely diverse sarcoma tumors may share a dependence on a particular kinase and that inhibiting this kinase may, therefore, be expected to be effective for all histological subtypes that are positive for this biomarker.

Aberrant regulation of the cell cycle is a hallmark of cancer, and multiple mechanisms contribute to the deregulation of the G1-to-S checkpoint [11, 12]. These mechanisms include amplification or mutation of the CDK4 and CDK6 genes, amplification of the genes encoding D-type cyclins and deletion or silencing of the *CDKN2A/B* gene, which encodes for the INK4 inhibitors p16^ink4a^ and p15^ink4b^ [13–15]. Additionally, the aberrant expression of growth factors or growth factor receptors and oncogenes can activate downstream signaling molecules that drive the expression of cyclin D1 [14]. Cell cycle deregulation is crucial for various oncogenic transformation processes, suggesting that many cancer cells depend on high CDK4/6 activity [16–21]. In contrast, the normal development of most tissues can occur in the absence of cyclin D-CDK4/6 complexes [22, 23]. Using strains of genetically modified mice, genetic studies have provided direct evidence for the role of CDK4 in tumor development. Mice lacking cyclin D1 were refractory to mammary tumor development induced by the ErbB2 oncogene, the ortholog of HER2, which is frequently overexpressed in human breast carcinomas [19, 24, 25]. Additionally, mice expressing a mutant form of cyclin D1 that binds to, but does not activate, CDK4 are resistant to erbB2-induced tumorigenesis. The ablation of CDK4 using siRNA in erbB2-induced mammary tumor cells eliminates their oncogenic properties [18]. The loss of CDK4 has also been implicated in the inability of KRasG12-induced lung tumors and c-Myc-induced skin tumors to develop [16, 21, 26]. CDK4/6 activity thus appears to represent a promising therapeutic target for cancer treatment [27–29].

Several highly selective inhibitors of CDK4 and CDK6 are currently being tested in phase II/III clinical trials against a variety of pRb-proficient chemotherapy-resistant cancers (http://ClinicalTrials.gov) [30, 31]. The broad-spectrum CKI flavopiridol displayed promising preclinical results in multiple tumor cell types [32–35], but it exhibited adverse effects and high toxicity in early-phase clinical trials [36]; furthermore, it did not meet expectations with regard to efficacy against most tumor types, with the exception of leukemia [34, 37, 38]. Palbociclib (PD0332991) is the first highly selective inhibitor of CDK4/6 to be tested and approved in humans for use in combination with letrozole for the treatment of postmenopausal women with estrogen receptor (ER)-positive human epidermal growth factor receptor 2 (HER2)-negative advanced breast cancer as an initial endocrine-based therapy for metastatic disease. Palbociclib exhibits an *in vitro* half-maximal inhibitory concentration (IC50) of 10–15 nM for CDK4/6, compared to 0.5 μM for CDK2 [39–41]. In fact, palbociclib has been tested in rabdomyosarcoma [42] and liposarcoma harboring elevated CDK4 expression [43, 44]. Recently, palbociclib has entered a phase II trial in patients with advanced CDK4-amplified or well differentiated liposarcoma [44]. Preclinical studies have demonstrated that palbociclib induces G1 arrest in pRb-positive cell lines and suppresses the growth of various xenografted tumors [31, 39–41, 45]. In different cancer models, treatment with PD0332991 not only exerts a cytostatic effect but also induces either the senescence or the apoptotic cell death of tumoral cells [46]. The only known mechanism of resistance to CDK4/6 inhibition is the loss of pRb function [16, 31, 45, 47]. However, other mechanisms such as p16^ink4a^ loss, cyclin D1 overexpression of elevated CDK2 expression have been proposed [31, 48, 49].

In the present work, we tested the suitability of CDK4 inhibition using palbociclib for sarcomas and explored possible markers of efficacy that are independent of the sarcoma tumor type. We found that tumor cells and patient-derived xenografts (PDXs) respond more strongly to a CDK inhibitor when they express high levels of CDK4 but exhibit resistance to the CDK inhibitor when they express high levels of p16^ink4a^.

## RESULTS

### Palbociclib induces senescence in sarcoma cell lines from different origins

To explore the effect of CDK4 inhibition, we used a panel of 10 low-passaged sarcoma cell lines generated directly from patient samples and 2 commercial cell lines of heterogeneous origin and different molecular karyotypes (Table 1) [53, 54, 57]. We treated these 12 sarcoma cell lines with different concentrations of palbociclib and obtained an IC50 of for each cell line. All responses were in the low μM range (Table 1). These values are higher than the reported in breast tumor cell lines [40, 58, 59].

**Table 1:** Characteristics of the sarcoma cell lines used and their response to palbociclib

Cell lines	Sarcoma type	Karyotype	Palbociclib IC50, μM
AW	Mixoid Liposarcoma	46,XY/47 +3/50%/50%	16.44 ± 1.62
AA	Leiomyosarcoma	55–63 < 3n > XXY,−3,−4,−8,i(8)(q10),add(8) (p23), der(11)t(5?;11)(q13;p15),−12,−13,−14,−16,der(1;7)(t(17;?) (p?;?),−18,−22, mar 1–3	26.63 ± 2.20
SW872	Liposarcoma	(5)t(5;?)(q31;?)1,dre(5)t(5;?)(q31;?)2,der(6)t(6;?)(q15:?),der(7)t(7;?)(q36;?),t(15q16q	15.79 ± 0.13
BD	Ewing's sarcoma	46, XY	23.07 ± 2.33
AX	Mixoid Liposarcoma	62–65, < 3n >, −3,−4,−5,+7,der(7)t(7;?),add(8) (p?),i(8)(q10),−9,−11,der(11)t(5;11),−13, add(14)(q32),−15,x2,−16,−18,+1–4mar cp5]	16.90 ± 0.20
BG	Myxofibrosarcoma	44–49.XY,der(1)t(1;3)(q12;p12).-3,−4,+6,+7 × 2, der(11) t(10;11;15),−13 × 2,−15, +der(16)t(16;?)(q12;?),del(17)(p11.2) +20[cp4]	20.96 ± 0.04
BC	MPNST	46, XX	12.43 ± 1.01
AZ	Fibrous tumor	46,XX/47,XX+8/50%/50%	17.42 ± 5.46
A673	Ewing's sarcoma	46∼47, XX, der(1)t(1;9)p36;q22),der(3)del(3)(p21) del(3)(q21), del(4)(q21q31), (5;8)(q33;q21),der(9)t(9;13)(q22;q14), +der(11)t(11;13)(p13;q14),der(13)t(1;13)p36;q14),der(13) t(11;13)(q13; q14)(11;22)(q24;q12),der(16)t(3;16)(q21?;q22),der(22)t(11;22) (q24;q12) [cp15].	8.95 ± 0.40
CE	Rhabdomyosarcoma	46, XY	15.72 ± 1.17
DA	MPNST	ND	25.9 ± 1.34
DD	Myxofibrosarcoma	ND	16.65 ± 0.15

The IC50 was calculated from the average of a minimum of 3 independent experiments performed on triplicate samples

**Figure 1:**
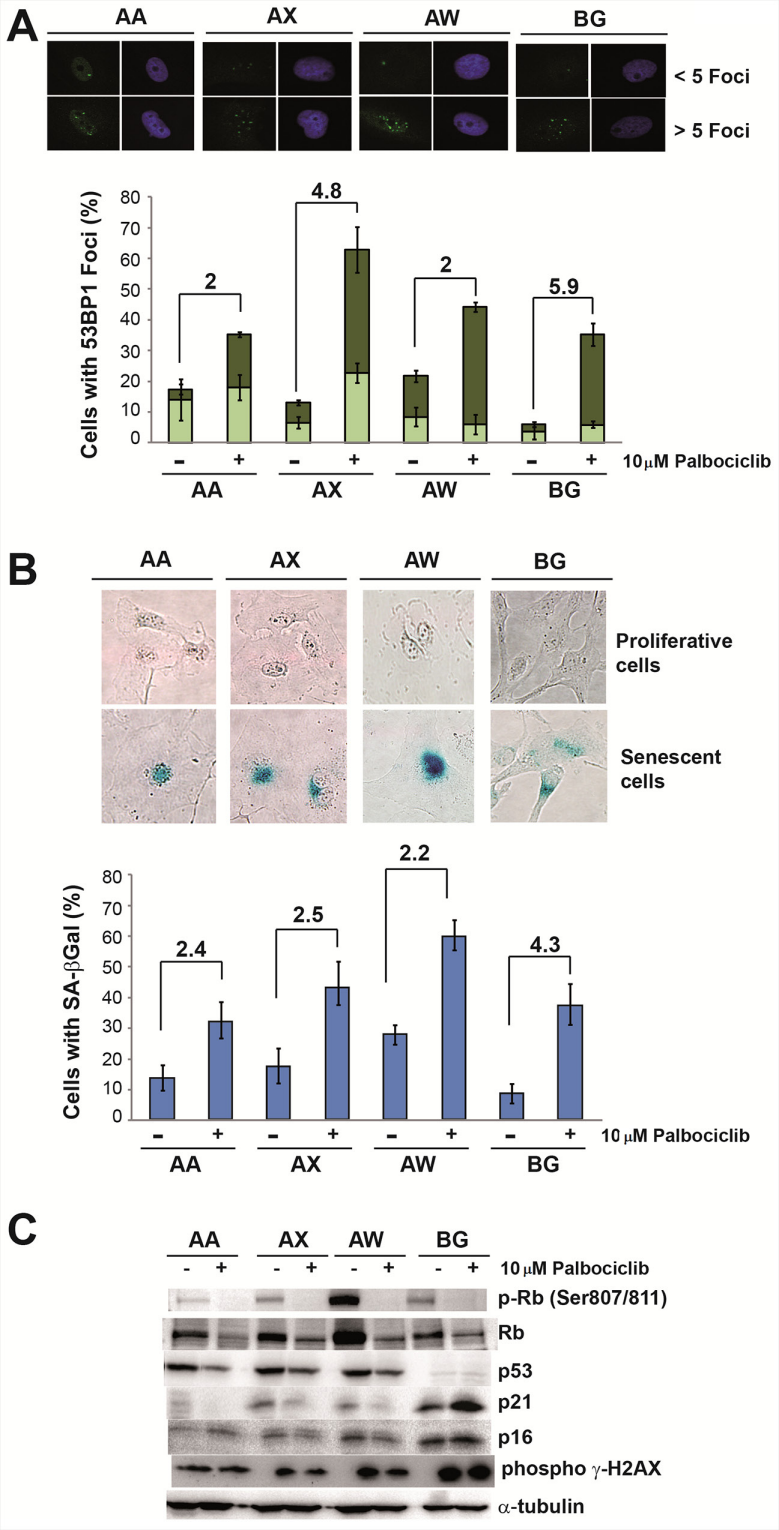
Palbociclib induces senescence markers in sarcoma cell lines **A.** Analysis of the levels of 53BP1 foci. Representative images (top) and quantification (bottom) of 53BP1 foci in the AA, AW, AX and BG sarcoma cell lines in the presence or absence of 10 μM palbociclib. **B.** Analysis of senescence-associated β-galactosidase (SA β-Gal) activity. Representative images (top) and quantification (bottom) of SA β-Gal activity in the AA, AW, AX and BG sarcoma cell lines in the presence or absence of 10 μM palbociclib. **C.** Western blot analysis of senescence markers in the AA, AW, AX and BG sarcoma cell lines in the presence or absence of 10 μM palbociclib.

To explore in detail the effect of this CDK inhibitor, we selected four different cell lines and measured the effect produced by palbociclib. We found that in all cell lines tested, palbociclib induced growth arrest at Go/G1 based on the detection of markers of cellular senescence (Figure 1). After 4 days of treatment, palbociclib induced the formation of nuclear foci containing the 53BP1 protein (Figure 1A), concomitant with strong SA β-Gal staining (Figure 1B) and reduced pRb phosphorylation (Figure 1C), as expected from the CDK4/6 inhibitory activity of palbociclib. However, we did not detect a significant change in the p16^ink4a^ levels. Moreover, in the BG cell line, the only cell line harboring wild type p53, treatment with palbociclib did not significantly alter p21^waf1^ protein expression (Figure 1C).

### Role of the CDK4 levels in the response of sarcoma cell lines to palbociclib

**Figure 2:**
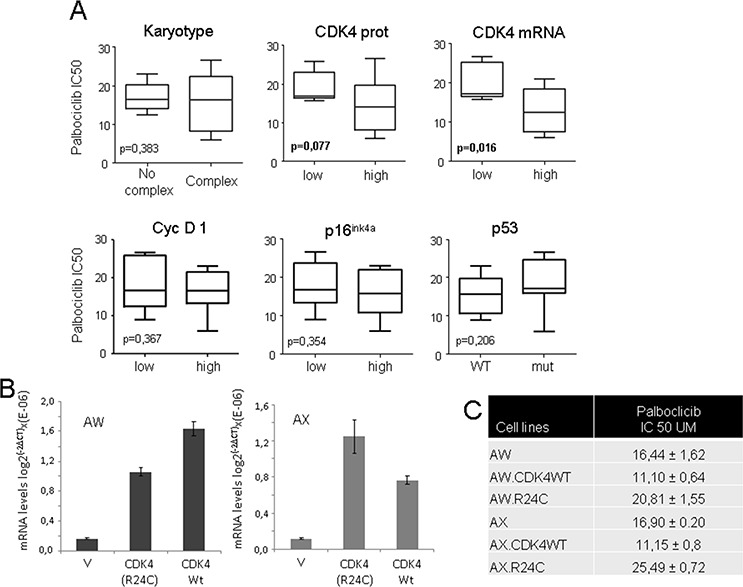
Correlation of the CDK4 expression level in sarcoma cell lines with their sensitivity to palbociclib **A.** Correlation of the levels of different cell cycle proteins with the cellular sensitivity to palbociclib. We analyzed the correlation (Student's *t* Test) of the presence or absence of mutant p53, p16^ink4a^, or cyclin D1, the CDK4 levels (as shown in table 2) or a complex karyotype (as shown in table 1) with the response to palbociclib. Increased sensitivity of palbociclib was related to high CDK4 levels at the mRNA (Student's *T* test, *p*= 0.017) or protein level (Student's *T* test, *p* = 0.077), but not to the cellular karyotype or p16ink4a, cyclin D1 or p53 status. **B.** AW and AX cells were transfected with empty vector (V) or a plasmid containing cDNA for wild type CDK4 (CDK4) or mutant CDK4-R24C (R24C). The graphs show the levels of mRNAs in the generated mass cultures after selection. **C.** IC50 of palbociclib for the AX and AW sarcoma cell lines and the generated lines overexpressing wild type CDK4 or mutant CDK4-R24C (R24C). The IC50 was calculated as the average of a minimum of 3 independent measurements performed on triplicate samples.

Although we examined a limited number of cell lines, which may limit the relevance of the findings, we next set up to explore whether the activity of palbociclib correlates with any molecular characteristics of the cells. Initially, we did not find any correlation of the palbociclib response with tumor type or the complexity of the karyotype (Figure 2A). It has been reported that in different cell models, the activity of palbociclib may be related to the p16^ink4a^, cyclin D1 or CDK4 levels. The levels of these factors were characterized in our cell lines (table 2, Supplementary figure S1), and we analyzed their correlation with the efficacy of palbociclib. We found that *in vitro*, the sensitivity of palbociclib is related to high CDK4 levels, either at the mRNA (Student's *T* test, *p* = 0.017) or the protein level (Student's *T* test, *p* = 0.077) (Figure 2A), but not to the levels of p16^ink4a^, cyclin D1 or p53 (Figure 2A).

**Table 2:** Presence or absence of certain cell cycle-related proteins in the cell line panel used in this study

Cell line	P53 mut	P16 INK4 (1)	Cyc D1(2)	CDK4 prot (3)	CDK4 mRNA (4)
AA	273H	0	0	1	0
AW	273H	0	0	0	0
AX	273H	0	0	0	0
AZ	WT	0	1	1	0
BD	WT	1	1	0	0
BC	WT	0	1	1	1
BG	Del	1	1	0	1
DA	273H	0	0	0	0
DD	175H	0	0	0	0
CE	WT	1	0	0	0
A673	WT	0	1	1	1
SW872	251N	1	0	0	1

(1): 1: presence of mRNA; 0: absence of mRNA

(2): 1: overexpression of protein; 0: absence or low levels of protein

(3): 1: high levels of protein; 0: normal levels of protein

(4): 1: high levels of mRNA; 0: normal levels of mRNA

To further explore the functional relevance of CDK4 levels to the efficacy of CDK inhibitors, we treated a panel of cell lines with flavopiridol and performed similar analyses (Supplementary Table S1). We found a similar correlation between the sensitivity of the cells to the CDK inhibitor and their levels of CDK4 (Supplementary Figure S3). As in palbociclib, we did not find any correlation between sensitivity to flavopiridol *in vitro* and the levels of p16^ink4a^ or cyclin D1 or the sarcoma type (Data not shown).

To study the functional relationship between the CDK4 levels and the cellular response to palbociclib, we overexpressed wild type CDK4 cDNA in 2 different sarcoma cell lines, AW and AX (Figure 2B). Because it has been reported that CDK4^R24C^, an active mutant of CDK4, is present in human tumors (athough not reported in sarcomas) and cannot be inhibited by members of the INK4 family, we decided to overexpress this mutant in these sarcoma cells (Figure 2B) in order to gain insight on palbociclib mechanism by exploring the effect of CDK4^R24C^ on its cellular response. An increased sensitivity to palbociclib was observed in cells overexpressing wild type CDK4, whereas the overexpression of the CDK4^R24C^ mutant increased the resistance of both sarcoma cell lines to palbociclib (Figure 2C).

**Figure 3:**
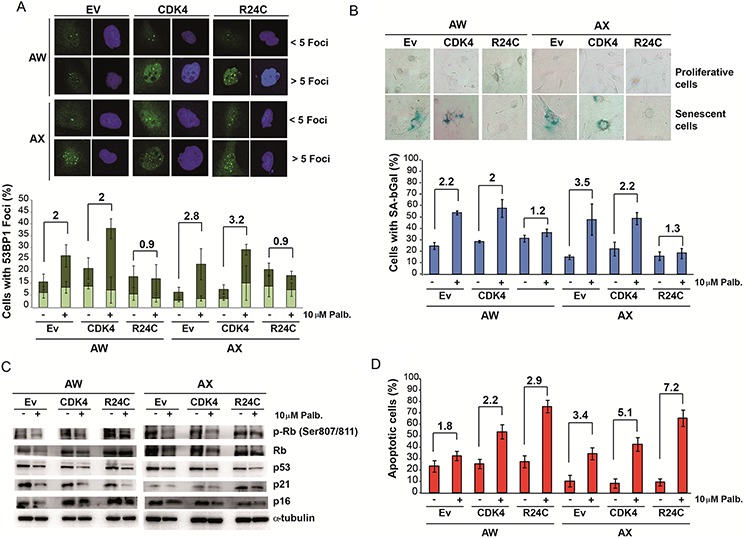
Physiological effect of palbociclib on cells overexpressing wild type CDK4 or INK4-insensitive mutant CDK4-R24C **A.** Analysis of the levels of 53BP1 foci. Representative images (top) and quantification (bottom) of 53BP1 foci in the AX and AW sarcoma cell lines transfected with empty vector (Ev) or a plasmid overexpressing CDK4 (CDK4) or mutant CDK4-R24C (R24C) after treatment with 10 μM palbociclib. **B.** Analysis of senescence-associated β-galactosidase (SA β-Gal) activity. Representative images (top) and quantification (bottom) of SA β-Gal activity in AX and AW sarcoma cells lines transfected with empty vector (Ev) or a plasmid overexpressing CDK4 (CDK4) or mutant CDK4-R24C (R24C) after treatment with 10 μM palbociclib. **C.** Western blot analysis of senescence markers in AX and AW sarcoma cells transfected with empty vector (Ev) or a plasmid overexpressing CDK4 (CDK4) or mutant CDK4-R24C (R24C) after treatment with 10 μM palbociclib. **D.** Percentage of apoptotic cells among AX and AW sarcoma cells transfected with empty vector (Ev) or a plasmid overexpressing CDK4 (CDK4) or mutant CDK4-R24C (R24C) after treatment with 10 μM palbociclib.

Next, we analyzed the effect of palbociclib on these engineered cell lines. We found that palbociclib induced cellular senescence in parental cells and in cells overexpressing wild type CDK4 (Figure 3A and 3B). The levels of senescence markers positively correlated with a reduction in the levels of phosphorylated pRb and an increase in the p16^ink4a^ and p21^cip1^ levels (Figure 3C). Alternatively, the induction of senescence markers was not observed in cells overexpressing the CDK4^R24C^ mutant; these cells preferentially entered apoptosis after palbociclib treatment (Figure 3D and Supplementary figure S3), although some apoptosis was also detected in the parental cells and in the cells overexpressing wild type CDK4 (Figure 3D and Supplementary figure S3).

### Effect of CDK4 inhibition on sarcoma PDXs

**Figure 4:**
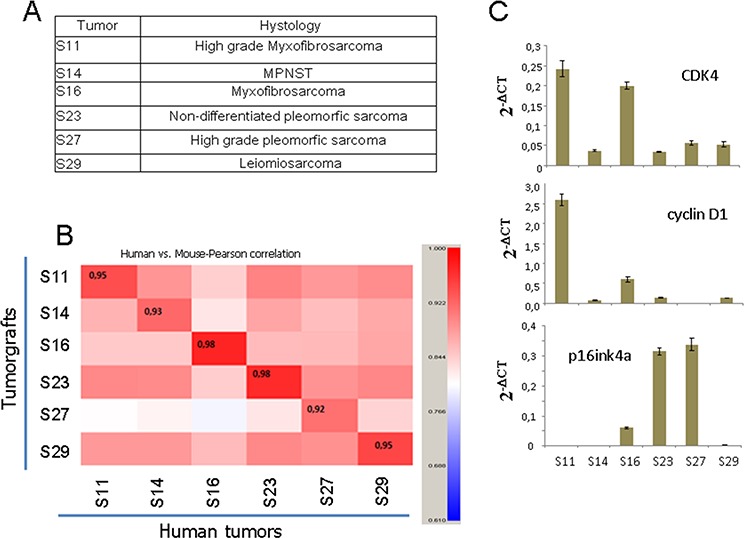
Characteristics of the PDXs used in this study **A.** Tumor histology of the 6 sarcoma PDX samples used. **B.** Comparison of the complete transcriptional profile of the original human tumor with the tumor graft grown in immunosuppressed mice. The number in the inset shows the correlation (0 to 1) between the original human tumor and the PDX used to explore the efficacy of palbociclib. **C.** The levels of CDK4, p16ink4 and cyclin D1 mRNA in the xenografted tumors used in this study.

**Figure 5:**
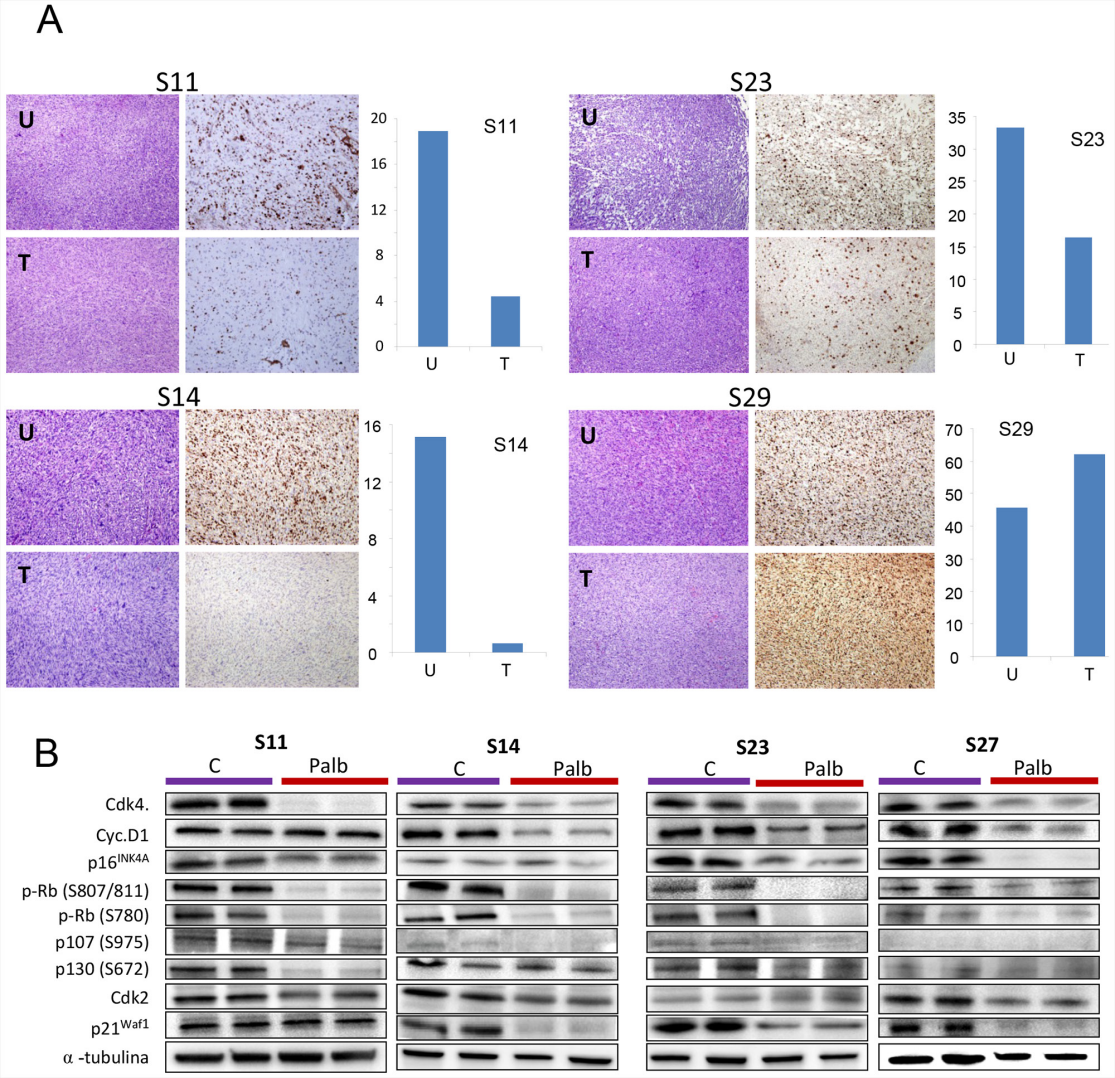
Effect of palbociclib on proliferation *in vivo* The sarcomas were subcutaneously engrafted and grown until all tumors reached 50 mm3 in volume. Then, the mice were treated with 2 mg/dose palbociclib for 3 weeks (5 days per week) via oral administration. The tumors were measured twice a week. The day after the final dose, the tumor-bearing mice treated with solvent (untreated) or palbociclib (treated) were sacrificed, and the tumors were analyzed for the expression of **A.** KI67, a marker of proliferation, **B.** several cell cycle related proteins by western blot. The tumors from the same initial engraft in both the treated or untreated groups were individually processed, and the levels of certain cell cycle proteins were analyzed. Western blots for CDK4, cyclin D1, phosphor-pRb (at Ser807/811; at S780), CDK2, p21waf1, p16ink4a, phospho-p130 (at S762), phospho-p107 (at S975) and a-tubulin (loading control) are shown. The figure shows 2 representative untreated and treated tumors from 4 models of the treated sarcomas.

To explore the effect of CDK4 inhibition *in vivo*, we used PDX models produced from a panel of different types of sarcoma. We selected 6 tumors from different tissue origins (Figure 4A). The comparison of the entire transcriptome of these tumor xenografts to the original human tumor showed between 93 and 98% similarity (Figure 4B), indicating that the PDX models were almost identical to the original human sarcomas. From these sarcomas, we tested 2 samples expressing high levels of CDK4 and cyclin D1 (S11 and S16), 2 samples expressing high p16^ink4a^ levels (S23 and S27), and two samples expressing normal levels of CDK4, low levels of cyclin D1 and no detectable p16^ink4a^ (S14 and S29) (Figure 4C). The sarcomas were subcutaneously engrafted and grown until all tumors reached 50 mm3 in volume. Then, the animals were treated with palbociclib. On the day after the final dose, mice harboring each tumor subtype treated with solvent (untreated) or with palbociclib (treated) were sacrificed, and the tumors analyzed for the expression of KI67, a marker of proliferation (Figure 5A). We found a variable effect of to palbociclib administration on the different sarcomas. Whereas some PDXs such as S11, S14 and S23 displayed a clear decrease in proliferation, others such as S29, did not show any effect of to palbociclib administration on KI67 expression (Figure 5A).

To explore the effect of palbociclib in these tumors we analyzed the response of cell cycle related proteins to palbociclib (Figure 5B). Thus, we collected the tumors the day after treatment from the same tumors and analyzed by western blot the the cell cycle relates proteins in tumors untreated and treated with palbociclib (Figure 5B). We can appreciate that palbociclib induces a decrease in all cell cycle related proteins, with a clear effect on pRb phosporylation in all models, either responding or not responding, as well as CDK4, confirming the activity of palbociclib in all models.

**Figure 6:**
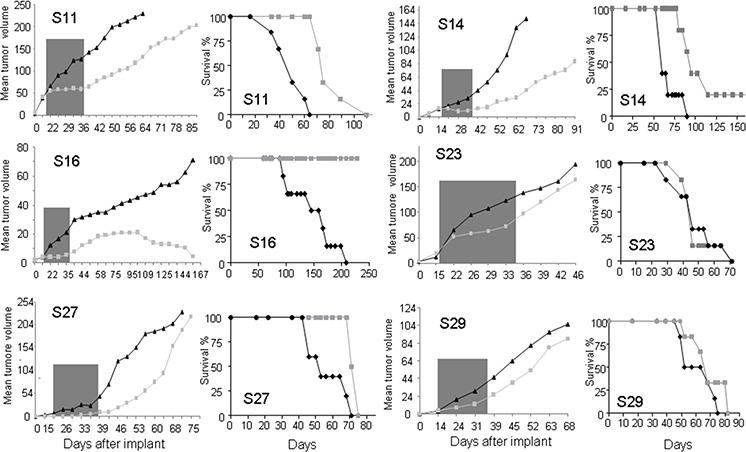
Effects of palbociclib on the sarcoma PDX models used in this study The sarcomas were subcutaneously engrafted and grown until all tumors reached 50 mm3 in volume. Then, the mice were treated with 2 mg/dose palbociclib for 3 weeks (5 days per week) via oral administration. The tumors were measured twice a week. Graphs showing the average size of all tumors (left) and the survival curve of all mice (right) are presented for each PDX model tested.

However, we observed different tumor behavior in response to the CDK4 inhibitor. Although we observed an initial inhibition of tumor growth in all tumors (Figure 6), after ending the treatment, the different sarcomas behaved very differently. The tumors expressing high CDK4 levels (S11 and S16) responded to the drug (figure 6). S16-derived tumors stop growing and even reduced in size in response to the treatment; as result, all treated mice survived. Alternatively, the S11-derived tumors responded to CDK4 inhibition by greatly delaying tumor growth; as a result, the treated mice survived almost twofold longer than the untreated mice (Figure 6). However, the two tumors expressing high p16^ink4a^ and normal CDK4 levels, (S23 and S27), showed some initial response to the CDK4 inhibitor, but after ending the treatment, tumor growth recovered to an even faster rate than that of the untreated tumors, and the survival of the treated mice was very similar to that of the untreated controls (Figure 6). Finally, the two tumors expressing normal CDK4 levels but low p16^ink4a^ levels showed heterogeneous responses. The MPNST-derived S14 PDXs responded to CDK4 inhibition with a clear reduction in tumor growth rate, and we even observed one tumor that disappeared in a mouse that survived. Alternatively, the S29-leiomiosarcoma responded initially to the CDK4 inhibitor, but after ending the treatment, tumor growth recovered, and the survival of the treated mice was only slightly longer than that of the untreated controls (Figure 6). We do not know whether a second round of treatment with palbociclib would have been more efficacious in these sarcomas because all of them appeared to respond to the first round of treatment; however, in some samples (S29), we did not observe a clear decrease in the expression of the proliferation marker KI67 (Figure 5). These data agree with the *in vitro* cellular data, suggesting that tumors expressing high CDK4 levels respond more strongly to palbociclib.

**Figure 7:**
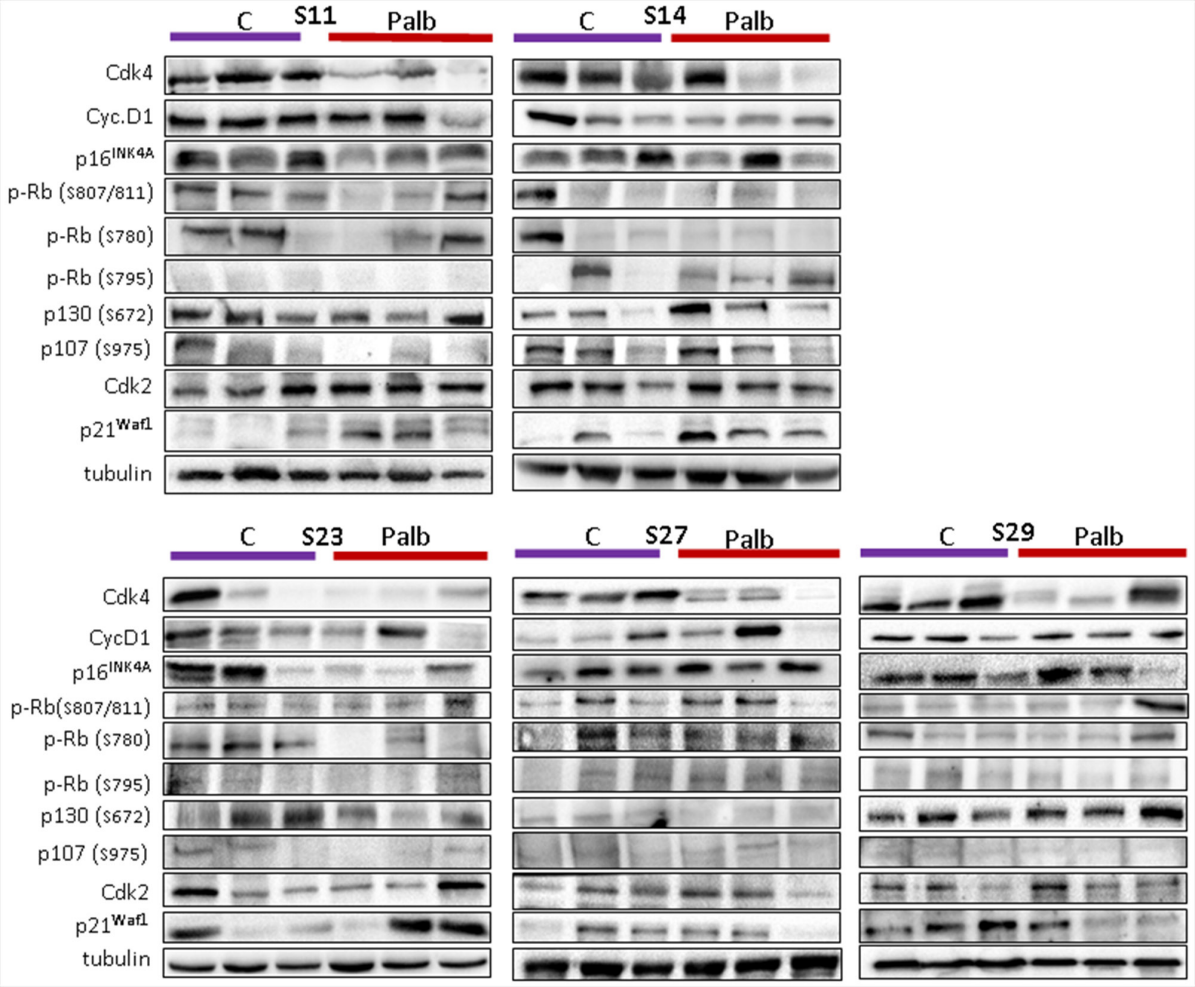
Levels of different cell cycle-related proteins in the sarcomas grown in untreated or treated mice At the end of the experiment, we collected all growing tumors and analyzed the levels of different proteins that may be involved in the response and/or the re-growth of the sarcoma. The tumors from the same initial engraft in both the treated or untreated groups were individually processed, and the levels of certain cell cycle proteins were analyzed. Western blots for CDK4, cyclin D1, phosphor-pRb (at Ser807/811; at S780; at S795), CDK2, p21waf1, p16ink4a, phospho-p130 (at S762), phospho-p107 (at S975) and a-tubulin (loading control) are shown. The figure shows 3 representative untreated and treated tumors from the 5 models in which we observed re-growth of the treated sarcomas.

At the end of the experiment, we collected all growing tumors and analyzed the levels of different proteins that may be involved in the response and/or the re-growth of the sarcoma. This could help us to determine whether molecular drift occurred in these tumors in response to the treatment and to identify possible drug resistance mechanisms. The tumors, either treated or untreated, from the same initial engraft were individually processed, and the levels of certain cell cycle proteins were analyzed (Figure 7 shows 3 representative untreated and treated tumors from the 5 models in which we detected re-growth of the treated sarcomas).

We observed that, in general, untreated tumors maintained or even increased their levels of CDK4, whereas most treated counterparts showed greatly reduced levels of this protein (Figure 7). We also observed some heterogeneity among the treated tumors, including some tumors that expressed high CDK4 levels, indicating the independent evolution of individual clones. We did not detect any association of palbociclib treatment with the behavior of phospho-pRb, measured at three independent sites, as high heterogeneity in the pRb levels was observed among clones. In our case, an increase in the pRb level appears not to be a determinant of treated tumor re-growth. Similar results were obtained in the site of CDK4 phosphorylation of p30, other pocket protein of pRb family. Similarly, we did not detect any association of the p16^ink4a^, cyclin D1 or CDK2 levels with the re-growth of treated tumors (Figure 7). Surprisingly, only an increase in p21^cip1^ expression was observed in the tumors in treated animals xenografted with responsive sarcomas (S14 and S11).

## DISCUSSION

The recent development of selective CDK4 inhibitors launched the first successful efforts to target the CDK4 pathway for cancer therapy. Several selective CDK4/6 inhibitors are currently in development, and these agents appear to be better tolerated than previous generations of CDK4 inhibitors. Three oral selective CDK4 inhibitors have entered clinical trials: palbociclib (PD0332991), LEE011, and LY2835219. CDK4 inhibitors display *in vitro* activity against a broad range of cancers and antitumor activity in patients with breast cancer, lymphoma, sarcoma, and other tumors [31, 60, 61]. Major efforts are underway to develop biomarkers of the treatment response and to understand the potential mechanisms underlying resistance to CDK4 inhibitors [31, 61].

To study the effect of CDK4 inhibition in sarcoma, a tumor type with few therapeutic approaches, we examined the effects of the CDK4 inhibitor palbociclib on sarcoma cell lines and PDX models. Treating these sarcoma tumors with the CDK4 inhibitor palbociclib revealed that tumors expressing high levels of CDK4 mRNA, but not those expressing low CDK4 and high p16^INK4a^ levels, responded to palbociclib. On the other hand, we have found that CDK4-R24C mutant increases resistance to the drug. Since the only known phenotype associated to the R24C mutation is the insensitivity to Ink4 inhibitors the mechanistic basis of this result is unclear. Palbociclib selectively binds the ATP binding site of the CDK4/6-cyclin D complex. It is possible that mutation in R24C of CDK4 slightly disturbs the ATP-binding pocket providing lower affinity of palbociclib for mutant CDK4, thus rendering this protein more resistant to its inhibition. We think that further crystallography studies with the mutant vs WT CDK4 proteins bound to the drug are necessary.

It has been reported that the efficacy of CDK4 inhibitors requires that the tumor cell express a normally functioning pRb. If there is loss of the intact pRb, then the G1 checkpoint becomes unrestricted, rendering the proliferation of malignant cells CDK4/6-independent and thus resistant to treatment with CDK4/6 inhibitors. However, some cell lines expressing an intact pRb exhibit CDK4/6 inhibition insensitivity, suggesting that other escape mechanisms exist [31]. Increased levels of p16^INK4a^ may serve as a marker of pRb deficiency [62, 63]. In our hands, *in vivo* studies suggest that high levels of p16ink4a are a marker of poor response to palbociclib, even in the presence of active pRb in the tumors as demonstrated by its phosphorylation as diverse sites (Figure 7).

In our *in vivo* study, a molecular pattern was related to the response to a CDK4 inhibitor. Whereas two sarcomas expressing high CDK4 levels responded (S11 and S16), the two sarcomas expressing high p16^INK4a^ and normal CDK4 levels showed a poor response and early recovery from treatment. However, among the tumors with normal CDK4 levels but low p16^INK4a^, there was some heterogeneity, as one tumor appeared to respond (MPNST-S14) but the other (Leiomiosarcoma-S29) showed similar levels of senescence markers to those in S14; however, the treatment response and the KI67 expression results were unclear. It is possible that leiomyosarcomas in general or this specific tumor are refractory to the drug *in vivo*. It is also possible that under possible limiting molecular conditions (normal CDK4 medium/low cyclin D and low p16^INK4a^), the response is worse than under conditions of the complete absence of p16^INK4a^. However, although we did not observe a correlation with tissue origin in the cell line results (data not shown), the observations that both responsive tumors originated from myxofibrosarcomas and that both clear nonresponders originated from pleomorphic sarcomas suggest that some tumor type-related effect on the treatment response is possible. Thus, additional experiments examining a large cohort of these tumor types are necessary. In line with the results of the responsive MPNST-S14 sample, it is worth mentioning that MPNSTs are sensitive to sorafenib, probably due to the inhibition of MEK and ERK, the suppression of cyclin D1, and the hypophosphorylation of pRb at CDK4-specific sites, resulting in cell cycle arrest [64]. The available evidence suggests that sorafenib, by inhibiting the MAPK pathway, inhibits CDK4 and that this drug may serve as a novel treatment for patients with MPNST.

As previously mentioned, increased levels of p16^INK4a^ may serve as a marker of pRb deficiency [62, 63]. However, high levels of p16^INK4a^ exist also in some tumors with wild type pRb as in those carrying HPV (E7 protein targets pRb), or that over express MDM2 [65], commonly co-amplified with CDK4 in sarcomas. However, in cells, we did not observe a significant correlation of the treatment response with high p16^INK4a^ levels (Figure 3), and we detected pRb in all tumors (Figures 5 and 7).

On the other hand, we did not observe a clear increase in pRb in tumors re-grown from treated tumors or an increase in p16^INK4a^ protein, indicating that other mechanisms of resistance also exist. Interestingly, we detected an increase in p21^waf1^ expression in tumors re-grown from treated tumors compared to the corresponding untreated tumors (figure 7). At this point, do not know whether this increase is mechanistically shutting down the cell cycle and inducing delayed tumor growth or only accompanies the remaining tumor, which re-grows during the recovery period.

Therefore, it appears that palbociclib can be applied to other sarcoma types, especially those expressing high CDK4 levels and low p16^INK4a^ levels. Similar data has been reported recently for dermatofibrosarcoma protuberans [46].

Increased CDK4 activity due to activating mutations, gene amplification, or the inhibition of CDK4-inhibitory signals via the silencing of p16^INK4a^ has been suggested as a tumor-initiating and/or tumor-promoting event in different tumor types. The amplification of CDK4, located on chromosome 12q13-14, has been reported in sarcomas, glioblastomas and breast cancers [66–69]. Activating CDK4 mutations have been reported in melanoma [70]. Both genomic events can lead to increased CDK4 signaling. Based on the accumulating evidence that CDK4 gene alterations are associated with the proliferation of different tumor types, CDK4 inhibitors are currently being studied in clinical trials.

Activating CDK4 mutations are less frequently reported in tumors than CDK4 gene amplification, and not reported in sarcoma tumors. Our data suggest that tumors carrying these mutants may not be suitable for treatment with palbociclib.

The amplification of chromosome 12q13-15 has been well documented in different sarcomas [66, 71–73]. Although it was originally thought that CDK4 is co-amplified with other Chr 12q13-15 genes, such as MDM2, it appears that different genes within this region are amplified dependent on the sarcoma subtype [74–76]. CDK4 gene amplification is commonly reported in atypical lipomatous tumors/well-differentiated liposarcomas, de-differentiated liposarcomas, and bone tumors. The overexpression of CDK4 was found to correspond to the gene amplification status and has been shown to be an especially useful marker for identifying well-differentiated and de-differentiated liposarcomas [77]. CDK4 amplification is correlated with a significantly poorer progression-free survival (PFS) and disease-free survival of liposarcoma [78]. Furthermore, in a small number of MPNSTs, or neurosarcomas, CDK4 gene amplification and subsequent increased CDK4 expression are significant predictors of poor patient survival [79].

Very little is known about whether genomic alterations in CDK4 can predict the tumor response to CDK inhibitors. Whereas the loss of p16^ink4a^ function has been consistently associated with sensitivity to CDK inhibitors, at least in preclinical settings, the association of increased CDK4 activity with increased drug sensitivity remains unclear. No completed study has demonstrated that activating CDK4 mutations affect the sensitivity of tumors or tumor cells to CDK inhibitors independently of 12q13-15 amplification. Preclinical and clinical studies of liposarcoma suggested that the amplification of CDK4 may predict an increased response to CDK4 inhibitors [43, 44, 80]. However, CDK4 amplification or overexpression predicted either CDK inhibitor resistance or showed no association with the drug response in tumor models of glioblastoma and melanoma [81]. Our data support a positive role of high CDK4 levels in the response to CDK4 inhibitors. There are ongoing trials designed to test the utility of CDK4 genomic alterations in predicting the tumor response to CDK inhibitors. It should be noted that CDK inhibitors are not expected to be effective for tumors lacking pRb or overexpressing wild-type p16^ink4a^ [61]. A phase II study demonstrated that the treatment of liposarcoma patients with palbociclib is associated with favorable PFS in tumors displaying pRb expression and CDK4 amplification [44]. Patients treated with palbociclib demonstrated 66% PFS, which significantly exceeded the primary study endpoint and included one partial response.

Alternatively, indirectly supporting the data suggesting CDK4 inhibition as suitable therapeutic strategy for sarcoma, in a phase 1 clinical trial, flavopiridol and doxorubicin combination therapy resulted in a disease control rate of 67% in progressive non-chemotherapy-responsive liposarcoma with likely CDK4 amplification [80]. The majority of patients exhibited stable disease at 12 and 24 weeks (PFS_12weeks_ and PFS_24weeks_), which is a favorable response rate in this patient population. *In vivo* sarcoma xenografts harboring CDK4 amplification also show significant responses to flavopiridol either as a single agent or in combination with doxorubicin.

In summary, our data support the efficacy of CDK4 inhibitors for sarcomas displaying increased CDK4 expression in general, particularly liposarcomas. Additionally, our results suggest that high levels of p16^ink4a^ may indicate a poor effect of these drugs on the tumor. Additionally, we show that tumors harboring active mutant CDK4^R24C^ may respond poorly to palbociclib but may be suitable targets for other CDK4 inhibitors such as flavopiridol. Our data reinforce previously published data on the suitability of CDK4 inhibition for cell cycle-dependent tumors and support the performance of new molecularly directed clinical trials for other types of sarcoma.

## MATERIALS AND METHODS

### Tumor samples

Tumor tissues were obtained via the surgical resection of sarcomas performed at Virgen del Rocio Hospital (Seville, Spain). All patients provided written informed consent according to a protocol approved by the local ethics committee (CEI 2013/PI002). The experiments were performed according to the European guidelines for laboratory animal care. This study was approved by the IBIS Institutional Animal Care and Use Committee.

### PDX generation

Sarcoma tissue samples were obtained from a single tumor area and were preserved in Dulbecco's modified Eagle's medium nutrient mixture/F10 (DMEM/F12; Sigma) containing 10% fetal bovine serum, penicillin, streptomycin and Amphotericin B (100 mg/ml each; Sigma). The samples were maintained for less than 2 hours in cell culture medium at room temperature before implantation. Each tissue was divided into 2 parts. One part was frozen, and the remaining part was cut into small fragments of 2–3 mm in diameter and used for subcutaneous implantation into 6-week-old Foxn1nu athymic nude female mice (Harlan Laboratories, Netherlands). Upon reaching a size of 1,500 mm3, the mice were euthanized, and the tumors were re-grown in a similar fashion to perform the indicated experiments.

### *In vivo* treatments

To initiate the experiments, each sample was xenografted into mice. Once the tumors reached 1500 mm3, the tumors were harvested, cut into 2 × 2 × 2 mm blocks and implanted. The experiments were performed using cohorts of 6 animals. Mice were randomly allocated to the drug-treated and control-treated (solvent only) groups, and once the tumor grew to 20 mm3, the mice were treated for 3 weeks (5 days/eek). The mice were monitored daily for signs of distress and were weighed twice a week. The tumor size was measured using a caliper according to the following equation: tumor volume = [length x width2]/2. The experiments were terminated when the tumor reached 1500 mm3. The drug (Palbociclib, PD0332991), was obtained from Pfizer, freshly prepared and orally administered. The concentration used in human is 125mg/dose. We have used higher doses in mice assuming a 70Kg average weight in humans. We have used 100 mg/kg in mice (equivalent to 2 mg/dose approximately, averaging 25 gr body weights each mice). This is equal or lower than the dose used in other xenograft studies but correspond to a 40 fold higher than the concentration used in humans. We have not found signs of toxicity.

### Western blot analyses

Western blot analyses were performed as previously described [50, 51]. Briefly, the cells were washed twice with PBS and lysed via sonication in lysis buffer (50 mM Tris-HCl, pH 7.5; 1% NP-40; 1 mM Na3VO4; 150 mM NaCl; 20 mM Na4P2O7; 100 mM NaF; 1% Na-deoxycholate; 0.1% SDS; 1 mM EDTA; phosphatase inhibitor cocktail (Sigma) and protease inhibitor cocktail (Sigma)). The samples were separated on 6–15% SDS-PAGE gels, transferred to nitrocellulose membranes (Protran BA83, Whatman) and immunostained. The following primary antibodies and dilutions were used: Anti-p21 [C-19] 1:200 (Santa Cruz, #sc-397), anti-p53 [FL-393] 1:200 (Santa Cruz, #sc-6243), Anti-p16 (M-156) 1:200 (Santa Cruz, #sc-1207), anti-pRb 1:500 (BD-Pharmingen), anti-phospho-pRb (Ser807/811) 1:1000 (Cell Signaling), anti-phospho-histone H2A.X (Ser139) 1:1000 (Millipore 05–636) and monoclonal anti-α-tubulin 1:1000 (Sigma 9026). Horseradish peroxidase-labeled rabbit anti-mouse (Amersham, diluted 1:3000) and goat anti-rabbit (Abcam, #6721, diluted 1:3000) secondary antibodies were used. The proteins were visualized using an ECL detection system (Amersham Biosciences).

### Immunostaining and confocal analysis of 53BP1 foci

Cells were seeded on glass cover slips and cultured for 16 h. Then, 10 μM palbociclib was added. After 48 h, cover slips were fixed in 4% paraformaldehyde for 5 min at room temperature, washed twice with PBS, permeabilized in 0.5% Triton X-100 in PBS for 5 min and washed twice more with PBS. The samples were incubated in blocking solution (PBS containing 3% bovine serum albumin) at 37°C for 1 hour, followed by incubation for 2 hours at room temperature in an anti-53BP1 antibody (Novus Biologicals, NB100-304) diluted 1:100. After washing with PBS, the cells were incubated in a species-specific Alexa 488-conjugated secondary antibody diluted 1:500 in blocking buffer for 1 hour at room temperature in the dark. The nuclei were counterstained with DAPI, and the slides were mounted using Prolong Gold Antifade reagent (Life Technologies). The samples were visualized under a confocal ultra-spectral microscope (Leica TCS-SP2-AOBS-UV) via sequential scanning of the emission channels. The mean fluorescence intensity was measured for a minimum of 300 cells per condition using Leica confocal imaging software. The plotted values represent the means (±SD) of each condition. Statistical significance was calculated using Student's *t*-test.

### Senescence-associated β-galactosidase activity

Senescence-associated (SA) β-galactosidase (β-Gal) activity was measured as previously described [52], except that the cells were incubated in 5-bromo-4-chloro-3-indolyl-β-D-galactopyranoside (XGal) at pH 5.5 to increase the sensitivity of the assay. The percentage of cells expressing SA β-Gal was quantified by inspecting > 300 cells in each o three independent experiments.

### KI67 staining

From each paraffin block, consecutive 5-μm tissue sections were cut, mounted, and dried on glass slides. The tissues were deparaffinized in xylol, followed by dehydration in graded alcohol solutions. Endogenous peroxidase activity was blocked using DAKO Blocking Solution (Agilent Technologies, United States) for 20 minutes at room temperature. Nonspecific protein binding was saturated using a phosphate-buffered saline (PBS) solution containing 10% fetal bovine serum, 1% bovine serum albumin and 0.3% Triton X-100 for 1 hour at room temperature. A mouse anti-human Ki-67 primary antibody (clone MIB-1; DAKO, Agilent technologies, United States) was used. The primary antibody was applied to tissue sections, and the slides were incubated overnight at 4°C. A secondary antibody (Envision/HRI Dako SM 802) was applied for 1 hour at room temperature, and the immunocomplexes were revealed using Substrate Buffer SM 803 DAB and the chromogen DM 827 (ENVISION FLEX DAKO). The tissues were counterstained with hematoxylin (Dako), rehydrated in a graded alcohol series, and mounted using coverslips.

### Quantitative mRNA determination

Total RNA was isolated via cell lysis in Qiazol reagent using an RNA Mini Kit (Qiagen, Inc.). First-strand cDNA synthesis was performed using 2.0 μg of RNA, random primers, a dNTP mix and Multiscribe Reverse Transcriptase in a total volume of 50 μl (High Capacity Transcription Kit, Applied Biosystems). The series of conditions used for RT-PCR were as follows: 10 min at 25°C, 120 min at 37°C, and 5 min at 95°C.

To measure human CDK4, cyclin D1 and p16 expression, real-time PCR was performed using an ABI 7900HT PCR system (Applied Biosystems). The qPCR reactions were performed in 384-well plates via TaqMan Gene Expression Assays (Applied Biosystems). Gadph expression was examined as internal control. The relative mRNA quantities were expressed as 2-βCt. Relative mRNA quantification and statistical analysis of qPCR data were conducted using RQ Manager 1.2.1 software (Applied Biosystems).

### Complementary RNA microarray assembly, hybridization, and analysis

To investigate the transcriptional profiles of the samples, we used a GeneChip PrimeView and a Human Gene Expression Array GeneChip 3 IVT PLUS Reagent Kit (Affymetrix, USA). Total RNA (100 ng) was used for a reverse transcription reaction in the presence of a poly(A) binding proteins to generate gene expression profiles from mRNA. RNA amplification is based upon linear amplification and employs T7 *in vitro* transcription (IVT) technology. In the second step, single-stranded cDNA is converted to double-stranded cDNA, which acts as a template for *in vitro* transcription. The reaction uses DNA polymerase and RNase H to simultaneously degrade the RNA and synthesize second-strand cDNA. Labeled complementary RNA (cRNA) was synthesized and amplified via IVT of the second-stranded cDNA template using T7 RNA polymerase, followed by purification, fragmentation, and hybridization to the GeneChip PrimeView array. Hybridization was performed in the GeneChip Hybridization Oven 645 instrument at a temperature to 45°C with rotation at 60 RPM for 16 hours, followed by washing in the GeneChip Fluidics Station 450 and scanning using a laser scanner (GeneChip Scanner 3000 7G, Affymetrix). Finally, the data were collected using Expression Console software.

### Human primary sarcoma cell lines and culture conditions

The sarcoma cell lines used in this study were previously characterized [53, 54]. The cells were maintained as a subconfluent monolayer in F-10 medium (Sigma) supplemented with 10% FBS, penicillin-streptomycin antibiotics (Sigma) and Fungizone (Amphotericin B, Sigma). Each cell line was cultured at 37°C and 95% humidity in 5% CO2 under conditions of O2 levels, culture medium and supplements indicated in the provider's instructions.

### Transfection

Subconfluent AW and AX cells were transfected using the Lipofectamine method (Effectene, Qiagen) with 0.4 μg of the empty mammalian expression plasmid pCMV6-neo or pCMV6-neo containing either the wild type CDK4 gene (Origene) or the R24C mutant CDK4 gene [55]. At 24 hours after transfection, the AW and AX cell lines were cultured in F-10 medium supplemented with 10% FBS and 0.8 mg/ml G418.

### Cytotoxicity assay

Palbociclib was freshly prepared as a 30 mM stock solution in sterilized deionized water for each experiment. Palbociclib was applied to a 96-well master plate at decreasing concentrations of 1/3, such that 300 μM was the highest concentration studied. The cell lines were seeded in 96-well plates (5,000–10,000 cells per well, depending on the cell size). At 24 hours after seeding, treatment was applied for 96 hours. Cell proliferation was determined by MTT assay and confirmed by crystal violet staining [56]. The IC50 was calculated using GraphPad Prism software.

### Apoptotic assay

Cells were cultured for 16 h, and then, 10 μM palbociclib was added. Untreated cells were used as negative controls to define the basal level of apoptotic and necrotic or dead cells. After 48 h, the cells were stained using the Annexin V-FITC apoptosis detection kit (Immunostep) according to the manufacturer's instructions. Briefly, the cells were washed in PBS and resuspended in Annexin-binding buffer, which included propidium iodide (PI) at a concentration of 1 × 10^6^ cells/ml. Then, Annexin V-FITC was added, and the cells were incubated for 15 minutes at room temperature (25°C) in the dark. After incubation, 400 μl of Annexin-binding buffer were added, and the cells were analyzed via flow cytometry within one hour using a BD FACS Canto II flow cytometer. The data were analyzed using BD FACS DIVA Software v 8.0.

## SUPPLEMENTARY FIGURES AND TABLE


