# Supplementary material for: Efficacy of CDK4 inhibition against sarcomas depends on their levels of CDK4 and p16ink4 mRNA
Source: Oncotarget. 2015 Oct 20;6(38):40557–74. doi: 10.18632/oncotarget.5829 (PMC4747352; doi:10.18632/oncotarget.5829)
Supplement: Supplementary file 1 [file oncotarget-06-40557-s001.pdf]

SUPPLEMENTARY FIGURES AND TABLES

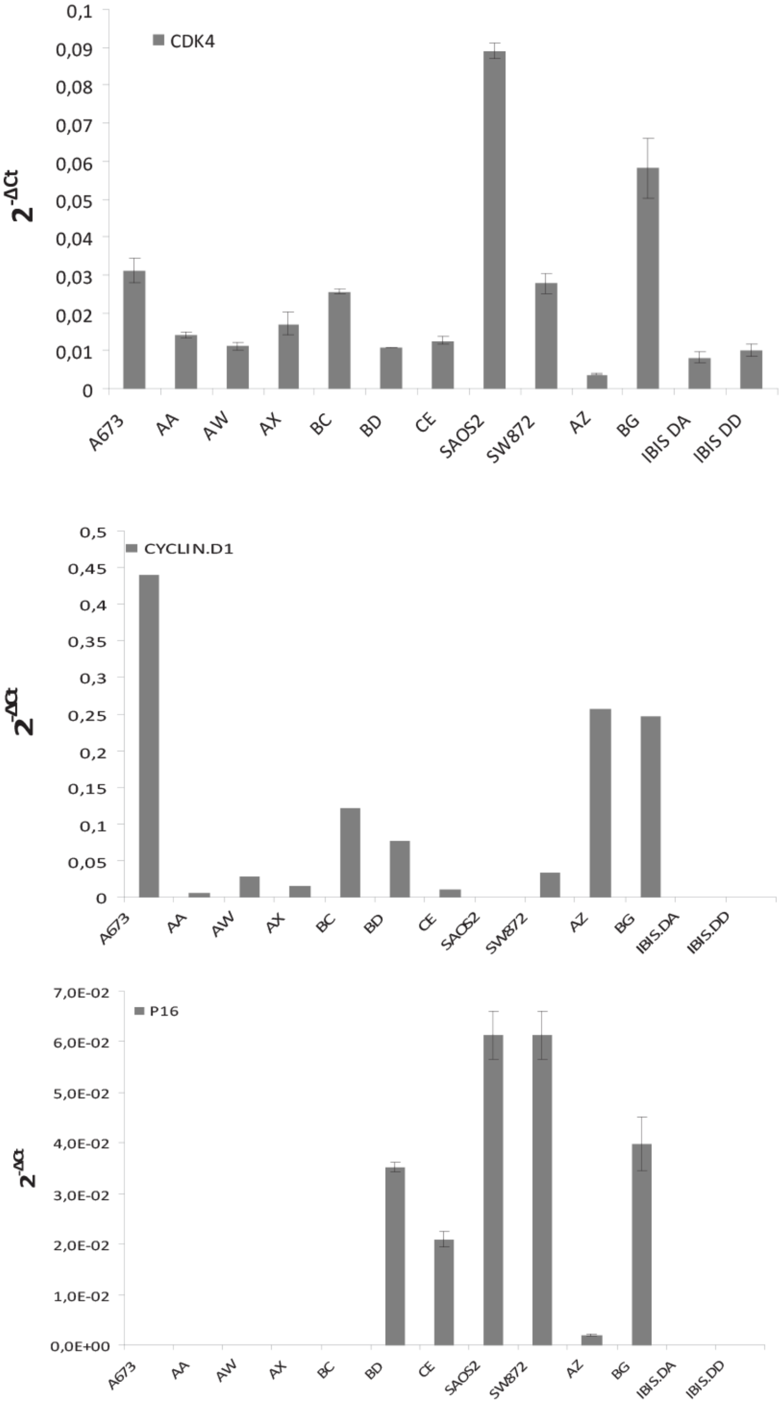

Supplementary Figure S1: The mRNA levels of CDK4, p16ink4 and cyclin D1 in the sarcoma cell lines used in this study. Levels were normalized to endogenous GAPDH.

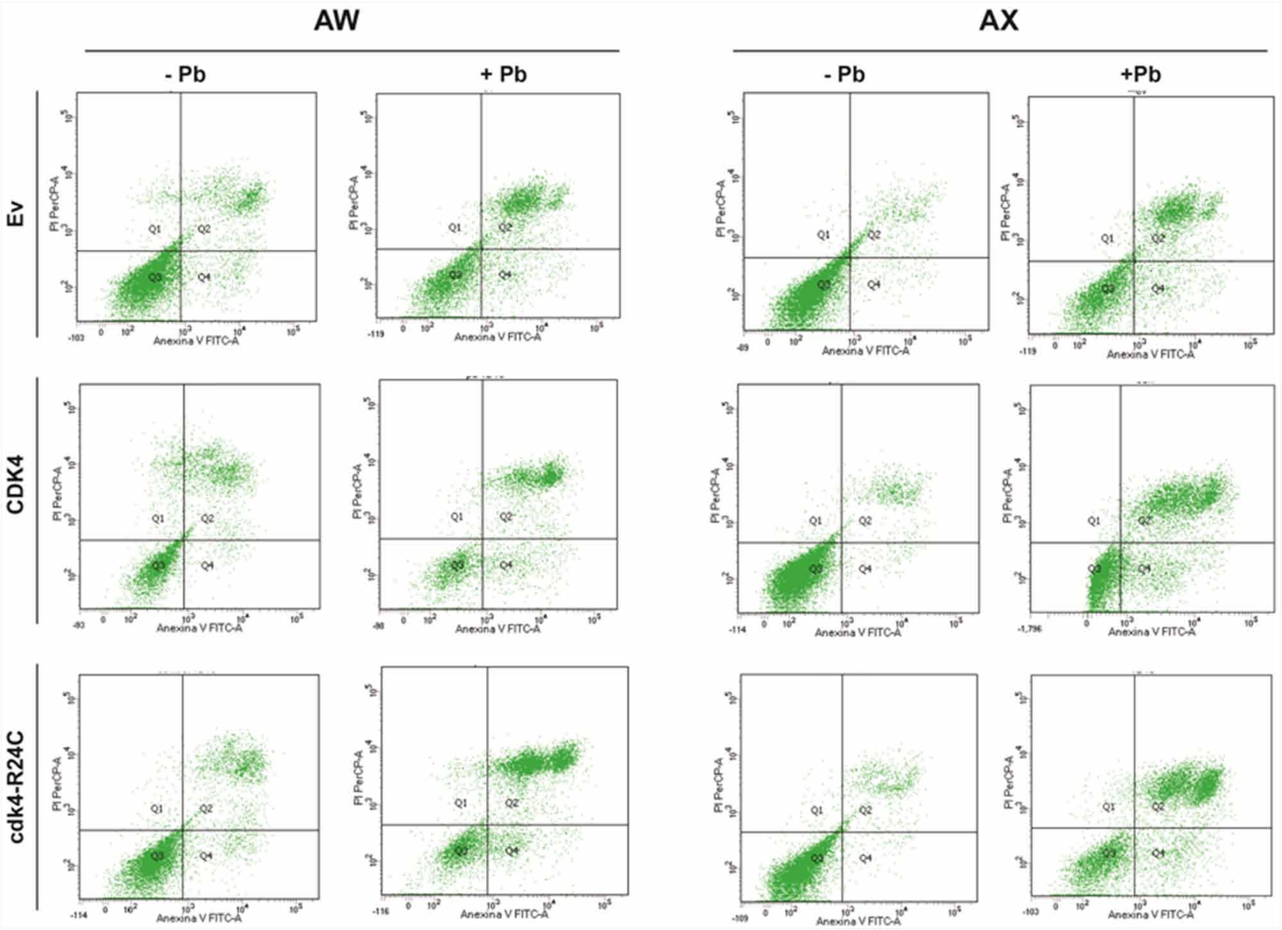

Supplementary Figure S2: Apoptotic assay using the AW and AX sarcoma cell lines. Representative scatter plots showing the levels of Annexin and propidium iodide (PI) staining via flow cytometry in AW sarcoma cells transfected with empty vector (Ev) or a plasmid overexpressing CDK4 (CDK4) or mutant CDK4-R24C (R24C) after treatment with 10 microM palbociclib. Cells positive for Annexin and PI (Q2) were considered as apoptotic.

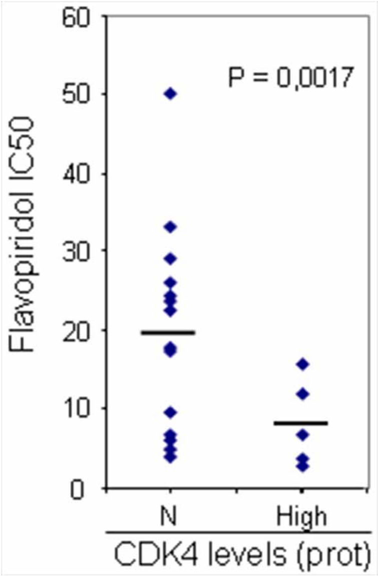

Supplementary Figure S3: The sensitivity of sarcoma cell lines to flavopiridol positively correlates with their CDK4 levels. We analyzed the correlation (Student's t Test) between the CDK4 protein levels in the sarcoma cell lines and their response to palbociclib.

Supplementary Table S1: Characteristics of the sarcoma cell lines used and their response to flavopiridol

CELL LINE	TUMOUR ORIGIN	Flavopiridol (nM)
**CNIO AW**	LIPOSARCOMA	29.08
**CNIO AX**	LIPOSARCOMA	17.85
**SW872**	LIPOSARCOMA	9.6
**1455**	LIPOSARCOMA	6.75
**CNIO AA**	LEYOMYOSARCOMA	6
**CNIO AY**	LEYOMYOSARCOMA	4.1
**CNIO AZ**	FIBROUS TUMOR	5
**CNIO** BC	MPNST	15.85
**CNIO BB**	MPNST	23.7
**A673**	EWING SARCOMA	12
**CNIO BJ**	OSTEOSARCOMA	6.8
**CNIO BF**	OSTEOSARCOMA	33.1
**CNIO BP**	OSTEOSARCOMA	26
**SAOS-2**	OSTEOSARCOMA	3.8
**CNIO BG**	MIXOID FIBROSARCOMA	24.5
**CNIO BM**	HIBERNOMA	> 10000
**CNIO BN**	FIBROHISTIOCITOMA	2.85
**CNIO CE**	RABDOMIOSARCOMA	17.5
**CNIO BI**	GIST	22.5

The IC50 was calculated as the average of a minimum of 3 independent experiments performed on triplicate samples
